# Magnetotransport Properties of Ferromagnetic Nanoparticles in a Semiconductor Matrix Studied by Precise Size-Selective Cluster Ion Beam Deposition

**DOI:** 10.3390/nano10112192

**Published:** 2020-11-03

**Authors:** Nicolas Gack, Gleb Iankevich, Cahit Benel, Robert Kruk, Di Wang, Horst Hahn, Thomas Reisinger

**Affiliations:** 1 Karlsruhe Institute of Technology, Institute of Nanotechnology, Hermann-von-Helmholtz-Platz 1, 76344 Eggenstein-Leopoldshafen, Germany; nicolas.gack@kit.edu (N.G.); gleb.andreevich@kit.edu (G.I.); cahitbenel@gmail.com (C.B.); robert.kruk@kit.edu (R.K.); di.wang@kit.edu (D.W.); horst.hahn@kit.edu (H.H.); 2 Karlsruhe Institute of Technology, Karlsruhe Nano Micro Facility, Hermann-von-Helmholtz-Platz 1, 76344 Eggenstein-Leopoldshafen, Germany; 3 Institute of Material Science, Technische Universität Darmstadt, Otto-Berndt-Str. 3, 64206 Darmstadt, Germany

**Keywords:** amorphous, germanium, semiconductor, iron, clusters, nanoparticles, nanocomposite, co-deposition, magnetoresistance, tunneling

## Abstract

The combination of magnetic and semiconducting properties in one material system has great potential for integration of emerging spintronics with conventional semiconductor technology. One standard route for the synthesis of magnetic semiconductors is doping of semiconductors with magnetic atoms. In many semiconductor–magnetic–dopant systems, the magnetic atoms form precipitates within the semiconducting matrix. An alternative and controlled way to realize such nanocomposite materials is the assembly by co-deposition of size-selected cluster ions and a semiconductor. Here we follow the latter approach to demonstrate that this fabrication route can be used to independently study the influence of cluster concentration and cluster size on magneto-transport properties. In this case we study Fe clusters composed of approximately 500 or 1000 atoms soft-landed into a thermally evaporated amorphous Ge matrix. The analysis of field and temperature dependent transport shows that tunneling processes affected by Coulomb blockade dominate at low temperatures. The nanocomposites show saturating tunneling magnetoresistance, additionally superimposed by at least one other effect not saturating upon the maximum applied field of 6 T. The nanocomposites’ resistivity and the observed tunneling magnetoresistance depend exponentially on the average distance between cluster surfaces. On the contrary, there is no notable influence of the cluster size on the tunneling magnetoresistance.

## 1. Introduction

In conventional micro- and nanoelectronics it is the electric field and consequently the electric current that determines the functional state of a device. The trend towards higher integration of circuits invariably leads to an increase of energy dissipation and leakage. The promise of spintronics is that limitations of this kind may be overcome by manipulating a spin degree of freedom in addition to electric current, or even replacing it [1,2]. Progress in the field has been enabled by the development of novel materials and device concepts, such as the discovery that multilayered or granular structures of ferromagnetic and non-magnetic metals exhibit giant magnetoresistance [3,4,5,6]. With this motivation in mind, we investigate a class of composite semiconducting magnetic material. Namely, we synthesize nanocomposite thin films by size-selective cluster-ion-beam deposition, with the aim of independently controlling the size and concentration of magnetic clusters in a semiconducting matrix.

Introducing ferromagnetism to a semiconductor while preserving its useful transport properties has been largely done by the development of dilute magnetic semiconductors (DMSs) [7]. A DMS is synthesized by substituting few percent of the semiconductor’s atoms by some transition metals with a finite magnetic moment. Ferromagnetism is then established by carrier mediated coupling of the dopant atomic moments.

Standard fabrication methods like molecular beam epitaxy, ion implantation, co-sputtering or pulsed laser deposition have been used to create DMSs out of elemental and multicomponent semiconductors embedding both 3d and 4f elements, e.g., Ge:Mn [8,9,10] and ZnO:Fe [11,12]. Ferromagnetic inclusions are not necessarily composed of the pure ferromagnetic dopant only, but they can also be a ferromagnetic alloy, e.g., ZnSnAs_2_:MnAs [13,14]. In some cases, thermite reactions can also be applied to synthesize DMSs [15]. 

One challenge is to achieve ferromagnetism above room temperature, a basic requirement in a wide range of applications. Recently, an amorphous metal-oxide magnetic semiconductor was synthesized from a ferromagnetic metallic glass that exhibits a Curie temperature higher than 600 K [16,17], the highest Curie temperature achieved in (Ga,Mn); a meanwhile well-established crystalline DMS is only 200 K [17,18]. In crystalline DMSs the increase in Curie temperature is limited by how many dopant atoms can dissolve in a semiconductor’s lattice whilst its crystal structure is preserved [17]. Often as a side effect, ferromagnetic inclusions can form. In these cases, dipole–dipole interaction between the inclusions increasingly determine the magnetic properties of the DMS. 

Isolated magnetic nanoparticles are interesting candidates for nanoscale metallic spintronic devices since they can exhibit much larger spin relaxation times compared to the bulk material. This is due to their large spin accumulation capability [19,20,21]. While spin valves can be implemented using layered structures exhibiting giant or tunneling magnetoresistance [3,4,22], both effects also occur in granular materials [5,6,23] and can thus be applied in spintronic devices [24]. Spin valve structures are additionally of relevance in superconducting circuits, where they can form part of Josephson junctions with a controllable phase shift [25,26]. These have potential applications in neuromorphic [27] and quantum computing, where pi-qubits are expected to exhibit reduced decoherence [28,29]. 

The combination of magnetic nanoparticles with semiconductors can result in intriguing phenomena like injection magnetoresistance [30,31], with promising applications in magnetic field sensing. As a further example, ferromagnetism in semiconductors has been applied to realize elements with a gate-tunable proximity magnetoresistance effect [32]. Takiguchi et al. [32] used a gated epitaxial bilayer structure of non-magnetic InAs, that forms a two-dimensional quantum well on top of a ferromagnetic (Ga,Fe)Sb semiconductor layer to this end. 

The method discussed in the present article, namely, directly embedding magnetic clusters by cluster ion beam deposition into a matrix material [33,34,35] is an alternative way to bring about magnetic properties in semiconductors. Because clusters are formed independently, before getting embedded by co-deposition within the matrix, the method allows for more precise control of the clusters’ composition, their size, and their average distance in the matrix. In some applications, it is desirable for the magnetic clusters to be small. For example, due to the thermal energy released when the moment of a superparamagnetic cluster aligns back to its easy axis after being aligned to an external field [36]. On the lower end, there is a limitation due to finite size effects. One example is the increasing fraction of misaligned surface moments [37,38]. Finally, as shown in the present article, co-deposition of magnetic clusters and semiconductors further extends the palette of materials, since even non-equilibrium compositions of elsewise at least partially miscible material combinations can be realized. 

In this article we investigate magnetoresistance of Fe_x_-Ge_m_ cluster–matrix nanocomposite films, prepared by size-selective low-energy cluster ion-beam deposition, as a function of cluster size, cluster concentration in the matrix and temperature. We provide an analysis of the dominant transport mechanisms in the films. The clusters, containing either 500 ± 50 (Fe_500_) or 1000 ± 100 atoms (Fe_1000_), are deposited from a size-filtered cluster ion beam and embedded into an amorphous Ge (a-Ge) matrix. Nanocomposite films with thicknesses from 17 nm to several hundred nanometers and Fe concentrations >15 at. % were grown. We measured resistivity and magnetoresistance of the films as a function of temperature. The latter is the change in resistivity due to an applied magnetic field—here up to $\mu_{0}H=6\;T$—and is defined as $\Delta \rho /\rho_{0}=\left(\rho \left(H\right)-\rho_{H=0}\right)/\rho_{H=0}$. The effect is on the order of 1% and composed of a saturating component at small fields and a field-dependent component extending to the highest fields. We identify the low field component as tunneling magnetoresistance. While the results are independent of particle size, we find a clear exponential correlation of tunneling magnetoresistance with resistivity and average nanoparticle surface-to-surface distance. 

## 2. Materials and Methods 

**Figure 1:**
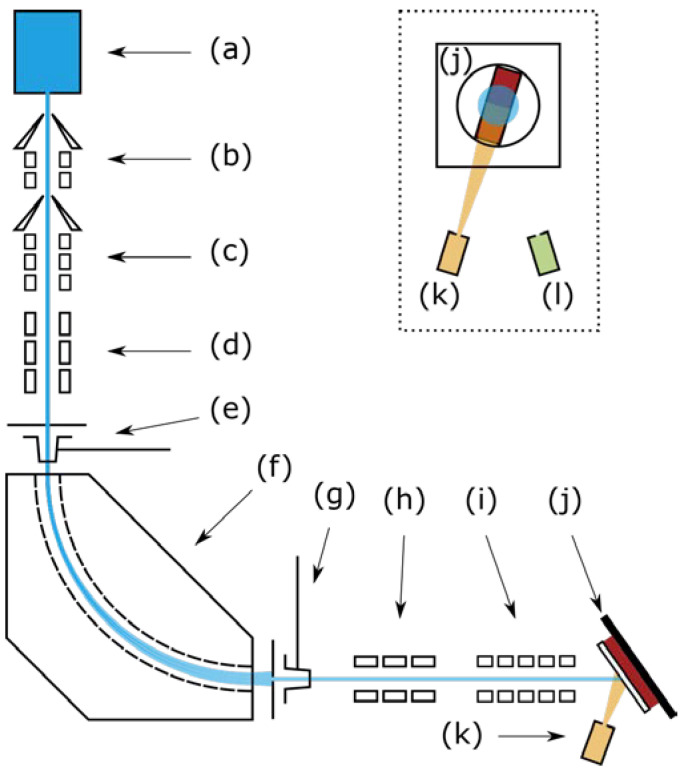
Sketch of the cluster ion beam deposition system. (**a**) Haberland-type inert-gas-condensation cluster-beam source, (**b**) 1st skimmer and acceleration lens set, (**c**) 2nd skimmer and acceleration lens set, (**d**) 1st quadrupole triplet, (**e**) 1st slit and Faraday cup, (**f**) 90° sector magnet, (**g**) 2nd slit and Faraday cup, (**h**) 2nd quadrupole triplet, (**i**) deceleration lens set and, (**j**) sample, (**k**) effusion cell and (**l**) triple electron beam evaporator. A third Faraday cup can be moved into the beam at the sample’s position (not shown). As indicated in the main sketch, effusion cell and triple e-beam evaporator are tilted by 70° to the horizontal cluster ion beam axis in vertical direction. As a compromise, the sample is set to an angle of 35° with respect to each incident beam. The inset depicts the ±17° tilt of the effusion cell and triple e-beam evaporator from the vertical direction. The slit mask (white) covering the sample chip (red) and its orientation is depicted there.

The cluster ion beam deposition (CIBD) system (in-house development) used in this study is capable of synthesis and deposition of size-selected clusters ranging from several hundred to more than one thousand atoms per cluster. In this work Fe clusters containing 500 ± 50% or 1000 ± 100% atoms (referred to as Fe_500_ and Fe_1000_ from here on) were deposited at a landing energy of 56 eV per cluster. Since the CIBD system is described in detail elsewhere [39,40,41] we will explain it here only in brief on the basis of Figure 1.

The CIBD system’s Haberland-type cluster source (a) [42] combines magnetron sputtering of a 2 inch target (Fe, Kurt J. Lesker Company, Jefferson Hills, PA, USA, N4 purity) with inert gas condensation (Ar and He mixture, Air Liquide, N6 purity) in a liquid nitrogen cooled aggregation chamber (AC).

Aggregated clusters exit the AC together with the inert gas into the vacuum chamber housing the AC through an adjustable iris. The iris’s opening and the sputter head’s adjustable distance from it allow to control both the pressure inside the AC and the time for cluster aggregation in the AC’s aggregation zone before being stopped by expansion into vacuum. High vacuum conditions are established via two skimmer-separated pumping stages (b), (c) right after the AC’s exit, where at first a turbopump with a nominal pumping speed of 1900 L/s counters the gas and nanoparticle load from the AC. In total, a nominal pumping speed of 4200 L/s is installed so that a remnant sputter gas pressure better than $10^{-7}\;\mathrm{mbar}$ during deposition and a base pressure better than 10^−8^ mbar are established in the deposition chamber when the AC is settled at cryogenic temperature. The set of applied source parameters depends on the cluster size aimed for and varied slightly from deposition to deposition. Exemplary ones are given in Table 1. 

Right behind the AC’s iris negatively charged clusters are attracted and accelerated in steps to a potential $U_{\mathrm{beam}}$ and collimated to a cluster ion beam by the two pumping stages’ skimmers (b), (c), a set of electrostatic acceleration lenses and an electrostatic quadrupole triplet (d). Positively charged clusters are deflected by the first skimmer. These and also the neutral ones are absorbed by the turbopumps. The cluster source itself is on ground potential. Because of collision processes it is unlikely for clusters to carry more than one elemental electric charge. A doubly charged cluster should have carried twice the amount of nominal atoms. However, from mass distribution scans the amount of double-sized clusters was found to be negligible. A cage-like metal tube shields the accelerated beam from ground potential and maintains its kinetic energy at $U_{\mathrm{beam}}$. The collimated cluster ion beam is directed into a 90° sector magnet (f) (Danfysik, Taastrup, Denmark, custom design, nominal radius 500 mm, maximum magnetic flux density 1.4 T, see Table 1). Adjustable slits at the entrance ((e), set to 5.3 mm) and exit of the sector magnet ((g), set to 18.0 mm) restrict the range of transmitted ion beam trajectories, which then correspond to a range of mass-to-charge ratio. The achieved mass resolution is better than 10%. Right behind each slit a Faraday cup can be moved into the beam to monitor and optimize the output of the source and the efficiency of the electrostatic lens system with the help of a picoamperemeter. Typical intensities of the size-filtered beam measured with the 2nd Faraday cup are included as $I_{\mathrm{Cl}}$ in Table 1. A second quadrupole triplet (h) recollimates the size-selected cluster ion beam after the sector magnet. Finally, a deceleration lens system (i) directs the beam onto a sample (j) installed inside the deposition chamber. The sample is at a potential of +56–57 V to establish soft landing conditions and was cooled to 135 K with liquid nitrogen to avoid cluster agglomeration. The films’ lateral dimensions on the sample chips were restricted using a laser-cut molybdenum deposition slit mask (1 mm×3.5 mm) as sketched in the inset of Figure 1. Exploiting the clusters’ single negative charge the picoamperemeter is used to also monitor the intensity of the beam hitting the sample in the CIBD system’s deposition chamber during deposition. Additionally, a third Faraday cup can be moved into the beam at the sample’s position in order to determine the beam density more accurately. However, a direct measurement of the cluster ion beam’s radial intensity profile is presently not possible. From the AC’s iris to the sample the cluster beam travels a distance of about 4 m.

**Figure 2:**
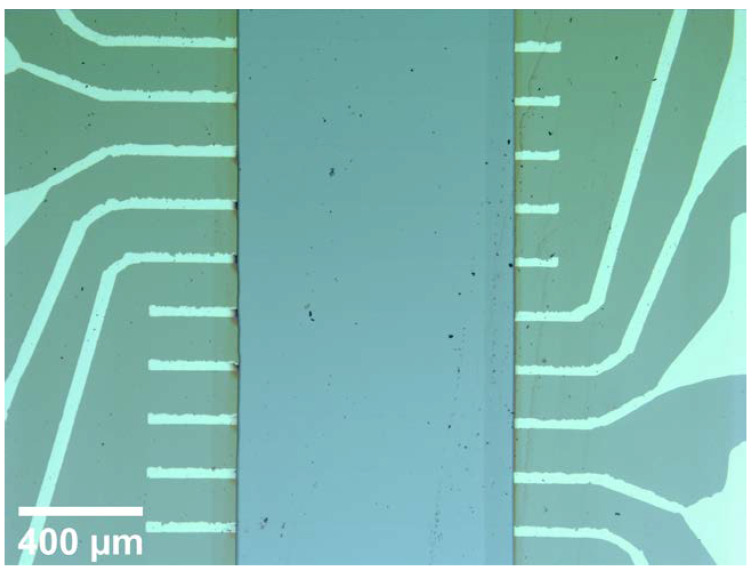
Optical micrograph of a sample with the nanocomposite film deposited across the contact line pattern.

An effusion cell (k) (CreaTec Fischer & Co. HTC, Erligheim, Germany) is located inside the deposition chamber of the CIBD system and provides a constant flux of Ge (MaTeck, Jülich, Germany, N5 purity, in alumina crucible). For this purpose, the Ge was heated to temperatures between 1100 °C and 1300 °C in an alumina crucible during deposition. The temperature was adjusted to obtain a deposition rate resulting in the desired cluster concentration together with the simultaneously deposited Fe clusters. The a-Ge matrix was grown with rates of up to 2 nm/min and monitored by a quartz crystal balance. Its output was calibrated using reference samples whose thickness was measured by XRR. Also, a triple electron beam evaporator (l) (Focus EFM 3, Si evaporated from a Ta crucible) is installed in the deposition chamber. While the cluster ions travel in a horizontal plane, the effusion cell and the triple evaporator are attached to the deposition chamber pointing almost vertically upward: They are rotated by 70° from the horizontal and laterally by ±17° to different sides with the rotation center equal to the sample position, as shown in the sketch of the CIBD system in Figure 1. In order to avoid columnar growth of the matrix due to shadowing effects [43], Fe-Ge samples were tilted by 35° from the horizontal for deposition. A side effect of the differing deposition angles is an offset between cluster ion beam, effusion cell and triple evaporator deposition areas due to the used deposition mask. In Figure 2 the offset between the two latter can be seen. The offset between matrix and cluster deposition was reduced to a minimum by mounting the chip under an angle of 17° in combination with a deposition mask that exhibited a correspondingly tilted slit. During deposition the samples were cooled to about 135 K by a constant flow of liquid nitrogen in order to avoid agglomeration of the deposited Fe clusters by reducing their surface mobility. The deposited Ge is amorphous, as expected for deposition temperatures below 450 °C [44]. We confirmed this by analyzing the magnetoresistive behavior of a reference film, which was negative from room temperature down to a lowest possible temperature of 220 K and followed a $\rho /\rho_{0}=-C\;{\left(\mu_{0}H\right)}^{n}$ dependence with temperature-dependent constant C and exponent n∈(0.5;1.0) [45,46,47]. 

**Figure 3:**
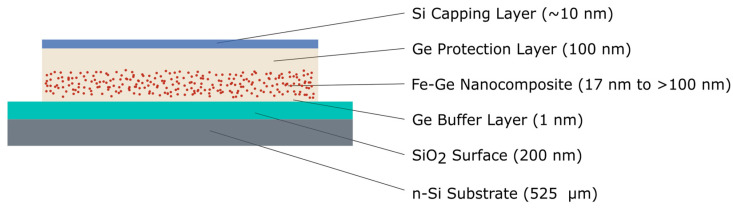
Cross-section through the layers of a co-deposited nanocomposite sample. The individual thicknesses are given in the drawing. Contact lines are not included in the sketch but can be seen in Figure 2.

Figure 3 shows a cross-section through a ready deposited sample.

Nanocomposite film stripes were deposited on the 200 nm thick SiO_2_ thermal oxide surface layer of a 5 mm×5 mm Si substrate (525 µm, {100}, P-doped, 10 Ω cm to 20 Ω cm). The nanocomposite films were sandwiched between a-Ge buffer and protection layers. Except one sample, all nanocomposite layers were thicker than 100 nm. The as-deposited a-Ge had a porous film structure and, hence, was prone to oxidation. For that reason, the sample film growth was finalized with a capping Si layer, deposited from the triple e-beam evaporator. 

Because of the protection layers it was easier to contact the nanocomposite film from underneath by a set of ten 40 µm wide Ti (5 nm)/Pt (20 nm) lines with contact pads, as can be seen in Figure 2. 

The contact pattern was deposited in advance by means of electron beam evaporation in another UHV system. Four adjacent lines were chosen for a four-wire measurement centered at the region most uniform in cluster concentration. 

As the presence of deposited clusters reduces the nanocomposites’ resistivity by orders of magnitude, the resistance of the buffer, protection and capping layers could be neglected. Measured two-wire resistances were always larger than 1.7 kΩ while a contact line’s resistance was about 300 Ω. Because noteworthy magnetoresistance effects only appeared at temperatures below 200 K and because the films’ semiconductor-like increase in resistance, the contact lines’ resistance did not turn out to be of significance. To confirm the concentration and its uniformity for each sample an EDX map was recorded after transport and magnetic measurements had been completed.

Resistance measurements as function of temperature and magnetic field were carried out in a liquid helium operated cryostat which is capable of precise temperature control and application of a magnetic field of up to 7 T (Quantum Design PPMS, San Diego, CA, USA). The PPMS provides internal excitation current sources with a minimum current in the nanoampere range. Each resistance was measured with at least four different excitation currents equally spaced in magnitude, the maximum being 200 nA at room temperature and decreased with temperature if necessary. To create a data point, for each current 25 readings in each current directions were averaged and calibrated to an internal calibration resistor by the PPMS (PPMS in AC drive and Standard calibration mode). To establish electric contact, the samples were bonded to a PPMS sample puck using a semi-automatic wire bonder. Samples were usually measured with the excitation current parallel to the applied field (longitudinal orientation). Some samples were additionally measured in transverse and perpendicular orientation. Standard zero-field/field cooled and magnetic hysteresis sequences were performed in longitudinal orientation in SQUID magnetometers (Quantum Design MPMS XL and VSM, San Diego, CA, USA).

We recorded detailed EDX maps across the part of the film where the voltage drop had been sensed (Zeiss Leo 1530, Jena, Germany, equipped with an Oxford Instruments X-Max N 50 detector, Abingdon, UK). This step was performed after the transport and magnetic measurements of a sample had been completed. The EDX map helped to confirm a sample’s local uniformity in concentration. Because of the difficulty of controlling the exact shape of the cluster ion focus on the sample and because of a moderate amount of charging effects, the cluster concentration deviated from the ideally expected top-hat shape. The so resulting cluster spot in the nanocomposite film had a diameter of about 1 mm to 2 mm with Gaussian-like concentration distribution. Therefore, it neither fully nor uniformly covered the 1 mm × 3.5 mm deposition area. In order to determine the atomic ratio of cluster vs. matrix material in the nanocomposite layer, we compensated the EDX determined atomic ratio of Fe and Ge using the amount of Ge deposited as buffer layer, as protection layer and within the nanocomposite layer as measured with the quartz crystal balance (see Appendix A). Furthermore, the amount of Fe in the film determined in this way was used to derive the local film thickness, which was needed to calculate sample resistivity. From the Fe concentration the mean particle separation (MPS) could be estimated. It is defined as the average distance between the surfaces of two neighboring clusters and is calculated by assuming a simple cubic order of the clusters in the matrix. 

## 3. Results

Fe-Ge nanocomposite films were analyzed and evaluated separately with regard to the following two aspects: Magnetoresistance and resistivity. For this purpose, Fe-Ge nanocomposite films with $c_{Fe}>15\;\mathrm{at}.\%$ were synthesized and studied. The samples and their basic properties are listed in Table 2. 

The room temperature resistivities of the samples varied over five orders of magnitude from $10^{-5}\;Ωm$ to $10^{0}\;Ωm$ depending on $c_{Fe}$ and exhibited negative magnetoresistance, which was for all cases below 1% in magnitude for magnetic fields up to 6 T. Except one sample (G5) all nanocomposite layers that did show magnetoresistance were thicker than 100 nm. Therefore, transport in the nanocomposite layers can be safely assumed to happen in all three dimensions. This does not hold for sample G5 because on average there are only two clusters stacked on top of each other between the buffer and the protection layer. It is also worth noting that all samples exhibit a mean particle separation less than the diameter of clusters embedded in the nanocomposite. 

The uncertainties of the absolute quantities given in Table 2 are governed by the XRR determined mass density of our a-Ge, the XRR determined crystal balance calibration and the EDX signal’s tampering by the protection and capping layers covering the nanocomposite. We estimated the following absolute errors to be $\Delta c_{Fe}=2\;\mathrm{at}.\%$, Δt=12 nm and ΔMPS=0.4 nm. The relative error of the resistivity is Δρ=13%. Since we deduced the tunneling magnetoresistances directly from the recorded curves we assume a relative error of ΔTMR=10% here. 

### 3.1. Magnetoresistance of Co-Deposited Fe-Ge Nanocomposite Films

**Figure 4:**
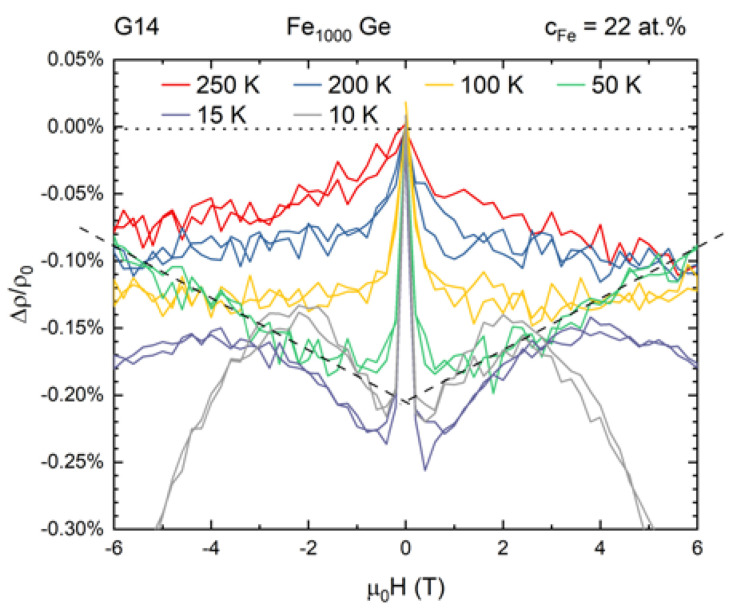
Set of magnetoresistance curves at different temperatures from sample G14. Intuitively, the magnetoresistance is a superposition of a sharp drop at low fields and a field-dependent component which is linear to good approximation above 40 K and non-linear below. The dashed lines approximate the high field magnetoresistance data at 50 K.

Within the examined range of Fe concentration samples showed magnetoresistive behavior up to a maximum concentration of about 30 at.%. In Figure 4 a representative set of magnetoresistance curves (relative magnetoresistive change $\Delta \rho /\rho_{0}$ vs. applied magnetic field $\mu_{0}H$) at different temperatures is presented for sample G14.

The detected magnetoresistance is a superposition of a sharp drop that saturates within low field range and a field-dependent component. The latter is linear to good approximation above 40 K and becomes significantly non-linear at lower temperatures. We separated the low field effect from each curve by extrapolating the approximated as linear field-dependent part of the curve graphically to $\mu_{0}H=0\;T$. As an example, corresponding dashed lines are added to Figure 4 for data at 50 K. 

**Figure A1:**
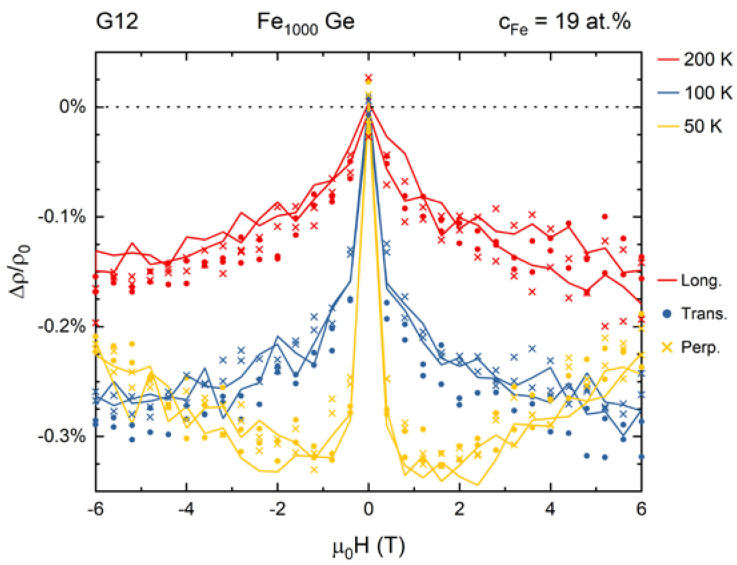
Magnetoresistance curves of sample G12 at three different temperatures in longitudinal (lines), transverse (circles) and perpendicular orientation (crosses). Evidently, the underlying magnetoresistance effects are isotropic.

Magnetoresistance curves were recorded up to fields of 6 T and in both field directions. For some samples the low field magnetoresistance was also measured at a higher field resolution. Transport measurements in longitudinal, transverse and perpendicular geometry showed that the observed magnetoresistance effects are isotropic as it is shown in Figure A1 in Appendix B. 

#### 3.1.1. Low Field Magnetoresistance

**Figure 5:**
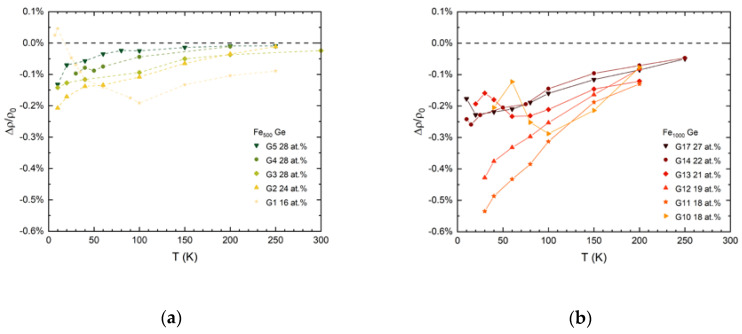
Low-field magnetoresistance vs. temperature of (**a**) Fe_500_-Ge and (**b**) Fe_1000_-Ge nanocomposite samples.

Separated from the high field magnetoresistance the magnitudes of the sharp drops are plotted versus temperature in Figure 5a for Fe_500_-Ge and in panel (b) for Fe_1000_-Ge samples.

The saturating low field magnetoresistance generally increases with decreasing temperature. It is larger in nanocomposite films which contain the larger cluster species and increases in magnitude with decreasing Fe concentration. However, the effect remains less than 1%. 

#### 3.1.2. High Field Magnetoresistance

**Figure 6:**
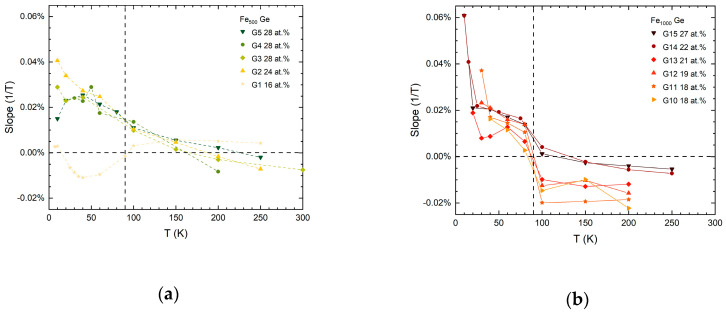
Slope of high field magnetoresistance vs. temperature of (**a**) Fe_500_-Ge and (**b**) Fe_1000_-Ge nanocomposite samples. Fe_1000_-Ge samples exhibit a jump to positive slopes around 90 K. A common trend is the increase of the slopes with decreasing temperature.

The slopes determined from the linear regression fits as indicated in Figure 4 are plotted in Figure 6 as a function of temperature.

For the Fe_1000_-Ge nanocomposite films, shown in Figure 6b, the slopes are initially negative for all samples starting from high temperatures and slopes increase as temperature is lowered. A change to positive slopes is observed at around 90 K, marked by the dashed vertical line. A similar trend is observed for the Fe_500_-Ge nanocomposite samples, as shown in Figure 6a. There, the jump to positive slopes is less pronounced, however. 

### 3.2. Resistivity of Co-Deposited Fe-Ge Nanocomposite Films

**Figure 7:**
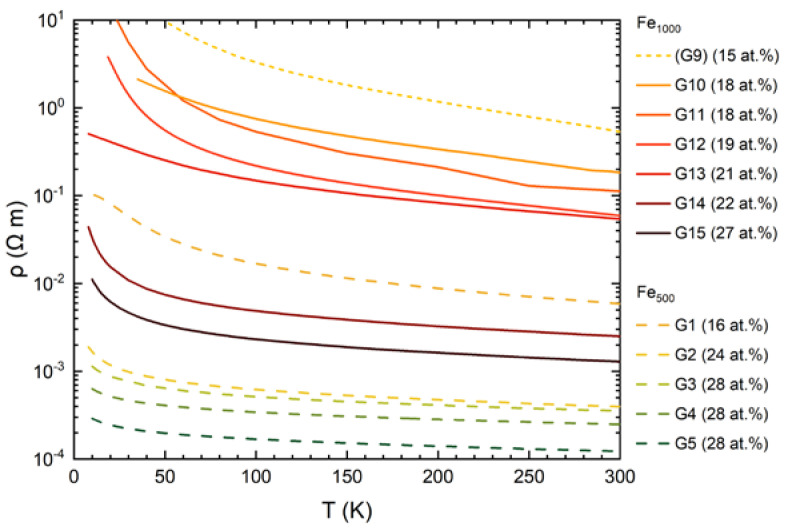
Resistivity vs. temperature of Fe_500_-Ge (dashed lines) and Fe_1000_-Ge (solid lines showing magnetoresistance, dotted line showing no magnetoresistance). Layers containing the smaller cluster species are lower in resistivity. Resistivity also decreases with increasing Fe cluster content. Not shown is the resistivity of an a-Ge reference sample, whose room temperature resistivity is $3\times 10^{2}\;\Omega m$ and from which it quickly increases with decreasing temperature.

Figure 7 presents resistivity data of the Fe-Ge nanocomposite samples as a function of temperature. Fe_500_-Ge data are plotted as dashed lines while solid lines represent Fe_1000_-Ge samples.

Data from sample G9, highest in resistivity and showing no magnetoresistance, are added as dotted line. In general, the resistivity increases approximately exponentially with decreasing temperature. The increase is faster than exponential below 100 K. All samples exhibit a linear I-V characteristic and hence ohmic contact behavior. The increase in resistivity is less in samples with higher Fe concentration and less for samples containing the smaller cluster species. For some samples containing Fe_1000_ clusters at low concentration the increase in resistance prohibited measurements at temperatures lower than 40 K. 

Within both of the two series of cluster species, resistivity increases with decreasing Fe content. For samples G13 and G10 the low temperature increase in resistance deviates from the behavior of the other samples. It is possible that the resistance of the films was shunted by some other conductor contaminating the film’s surface. One possible source is remaining conductive tape that was needed to ground the Pt contact lines during deposition. Although care was taken to remove it using solvents we cannot completely rule it out. A different reason may be slightly increased concentrations of clusters close to the edge of the film due to a small amount of charging on the mask or substrate surface. The flattening resistivity of G1 at the lowest temperatures maybe due to the same effects. 

### 3.3. Properties Related to the Superparamagnetic Nanoparticles

A TEM study was performed to characterize the size distribution of the deposited clusters and to examine if clusters are stable within the a-Ge matrix. The level of agglomeration of the clusters is visible in the micrographs, but it is difficult to transfer the result to the other samples because of the different substrate surface (carbon coated grid). In order to further prove the existence of the clusters inside the a-Ge matrix, we performed SQUID magnetometer measurements of all samples, confirming the superparamagnetic nature of the nanoparticle ensemble. 

#### 3.3.1. Analysis of Fe_1000_ TEM Grid Samples 

**Figure 8:**
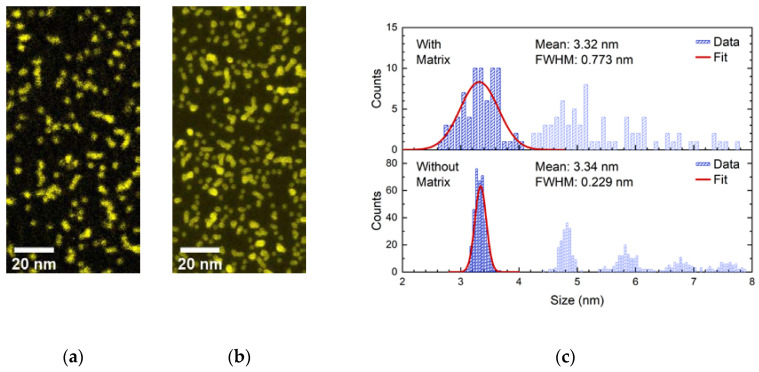
(**a**) Fe map of a 5 nm thin (quasi-2D) Fe_1000_-Ge nanocomposite containing about 7 vol.% of Fe clusters, acquired by means of EFTEM. (**b**) Scanning TEM micrograph of clusters deposited without Ge matrix on a thin carbon film. In both images isolated chains of clusters are clearly visible. Both images only show sections of the recorded images. (**c**) Particle size distributions extracted from the full images. The upper (lower) distribution corresponds to clusters co-deposited with (deposited without) Ge matrix.

In Figure 8a,b we present micrographs of two Fe_1000_-Ge nanocomposite thin films that were deposited onto carbon coated TEM grids. About 5 nm of Ge were co-deposited with the Fe clusters for the sample shown in panel (a), which resulted in an Fe cluster concentration of about 7 vol.% here. The micrograph was observed by acquiring an elemental map of Fe by means of energy-filtered TEM (EFTEM) using a three-window method at the Fe L_2,3_-edge. The other sample was deposited without Ge matrix; the corresponding image (b) shows a scanning TEM micrograph. To minimize agglomeration both samples were cooled with liquid nitrogen during deposition. In both images it is clearly visible that some clusters remain isolated while some other clusters agglomerate and form chains because of the random deposition from the cluster ion beam. Both samples are thin enough to be interpreted as a two-dimensional distribution of clusters, although it is possible that clusters apparently agglomerating in panel (a) remain disconnected. Moreover, graph (a) shows that the Fe clusters preserve their near spherical shape in the Ge matrix and mostly do not merge into larger particles or precipitates during or after film deposition. A size analysis of the particles in the matrix co-deposited TEM grid sample is shown in the upper plot of Figure 8c.

It yielded a slightly larger mean cluster size (3.3 nm) than the one given in Table 1. The lower plot shows the size distribution determined from the TEM grid sample carrying clusters deposited without Ge matrix. The average size of isolated clusters determined from each TEM grid sample match perfectly. Moreover, the latter distribution’s FWHM confirms that the CIBD system’s size selectivity is better than 10%. Graphs (a) and (b) of Figure 8 only show sections of the recorded micrographs. The size analyses were each executed on the full micrograph, that is, on areas that were larger than implied by the presented images. 

The TEM grid samples had to be continuously kept under inert gas atmosphere. For this reason, the TEM grid samples were transferred into a glove box while contained in an evacuated, UHV compatible transfer chamber. In the glove box they were loaded onto a Gatan 648 transfer holder under Ar atmosphere. However, HRTEM analysis of some isolated clusters with a crystalline structure in their cores suggests that of Fe_3_O_4_ to have formed. This indicates that, despite the met precautions, oxidation cannot be completely avoided. Therefore, oxidation of the Fe clusters is likely to be one reason for the increased cluster diameter observed here. Another reason may be a deformation towards an oblate ellipsoidal shape in the moment a deposited cluster hits the surface of a sample film. 

#### 3.3.2. Magnetization Measurements

**Figure 9:**
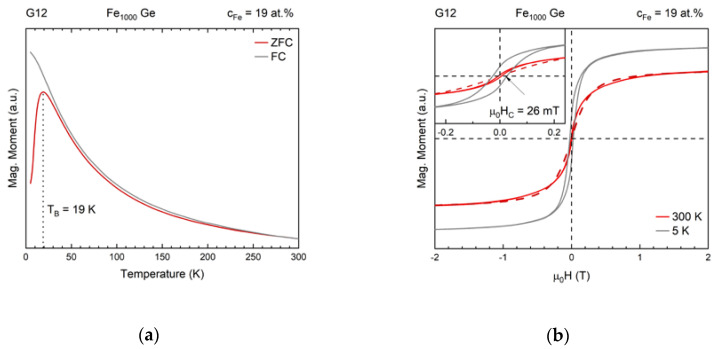
Magnetization measurements of sample G12. (**a**) ZFC/FC data plot reveals a maximum ZFC magnetization at 19 K. (**b**) Magnetization curves at 300 K and 5 K. Inset: The latter temperature reveals the film’s blocked (ferromagnetic) state due to a hysteresis in magnetization while the former reveals an anhysteretic dependence as expected in the superparamagnetic temperature range. The dashed line is a Langevin function that approximates the magnetization curve at 300 K.

The Fe clusters form a superparamagnetic ensemble in each sample, with the strength of interaction between their moments depending on their average separation. The superparamagnetic nature of each sample was confirmed by performing zero-field cooled/field cooled (ZFC/FC) measurements in a longitudinal field of 20 mT using a SQUID magnetometer. Results for sample G12 are shown exemplarily in Figure 9a.

The appearance of a maximum in magnetization in the ZFC curve and a coincidence of ZFC and FC curve above the maximum together with the lack of hysteresis at room temperature are evidence for the existence of a superparamagnetic state and, hence, individual, separated clusters in the film. The small disparity between ZFC and FC in the superparamagnetic temperature range is likely due to a small amount of clusters deposited outside of the matrix, which agglomerated on the contact lines because of charging effects. These agglomerates are visible as dark spots at the left hand edge of the example film shown in the micrograph Figure 2. 

**Figure 10:**
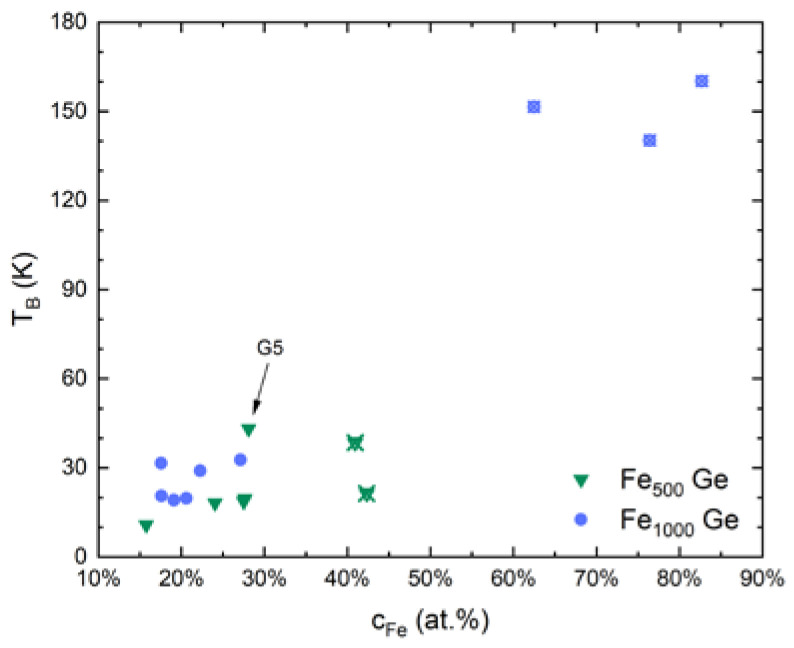
Blocking temperature of all samples (blue circles: Fe_1000_-Ge, green triangles: Fe_500_-Ge) that exhibit magnetoresistance (solid symbols). Some samples not showing magnetoresistance (crossed symbols) are added to the graph despite not being listed in Table 1. Disregarding the highlighted thin sample G5, the blocking temperature increases with concentration and is larger for the nanocomposites containing the larger cluster species.

While the cluster concentration was uniform within the regions of the films used for transport measurements, the shape of the cluster ion beam resulted in a concentration gradient away from the center of the cluster spot. Due to the varying degree of interaction among clusters and a varying degree of agglomeration, blocking happens across a temperature range instead of at a well-defined temperature. For the presented nanocomposite samples the maxima of the ZFC curves generally lie between 10 K and 40 K. Disregarding the thin sample G5, a slight increase of the blocking temperature with increasing Fe concentration was observed for the samples exhibiting magnetoresistance. On average, Fe_1000_-Ge samples possess a higher blocking temperatures (25 K) than the Fe_500_-Ge ones (17 K) as expected and as can be seen in Figure 10.

For samples above the percolation threshold much higher blocking temperatures were observed. Following the standard blocking temperature definition $VK_{1}/k_{B}T_{B}=\ln\left(\tau /\tau_{0}\right)\approx 25$ [48] with cluster volume V a single Fe_1000_ cluster’s blocking temperature can be estimated with the bulk magnetocrystalline anisotropy constant of Fe at liquid helium temperature $K_{1}=53.1\times 10^{3}\;J/m^{3}$ [49] to be about 2 K. The discrepancy with the observed blocking temperatures can be attributed to dipole interactions between clusters or for example a dominating surface anisotropy. 

In Figure 9b magnetization curves at 5 K and 300 K of sample G12 are shown. At 5 K, well below the sample’s blocking temperature, ferromagnetic behavior, indicated by the required coercive field to zero the magnetization, is observed (see inset) as expected. At 300 K the coercive field strength remains within measurement uncertainty. In addition, at 300 K the saturation magnetization of a sample is lower than when measured at 5 K, as expected from the thermal fluctuation of the superparamagnetic moments of the clusters. This presented behavior of the magnetization was observed for all samples. 

Additionally, in Figure 9b, as a crude approximation a simple Langevin model for the magnetization curve at 300 K is plotted as a dashed line. Using a magnetic moment of about $4200{\;\mu }_{B}$ in the model corresponds best to the data. We know the magnetic moment of bulk α-Fe to be $2.2{\;\mu }_{B}$ and can therefore conclude that the number of atoms contributing to an individual superparamagnetic moment is about twice the number of atoms in the as-deposited particles. This discrepancy arises both from agglomeration (a certain number of dimers exist as can be deduced to some extent from the TEM analysis) and the dipolar interaction among the nanoparticles. 

**Figure 11:**
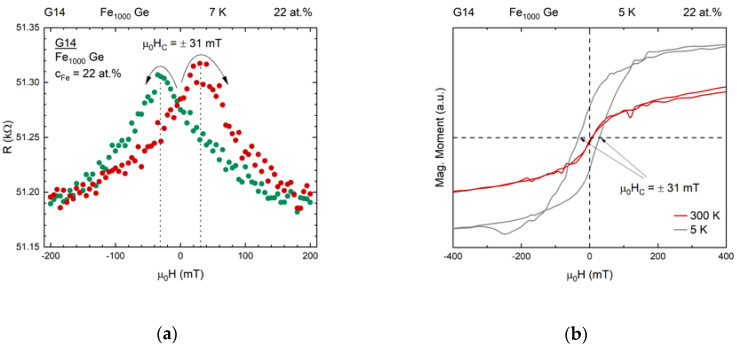
(**a**) Absolute resistance vs. magnetic field of sample G14 recorded at 7 K. The arrows and the color coding indicate the sweeping direction of the field. The points of maximum resistance at $\mu_{0}H=\pm 31\;\mathrm{mT}$ are passed after zero-crossing. (**b**) Magnetization vs. magnetic field shows hysteretic dependence at 5 K, again with a coercive field $\mu_{0}H_{C}=\pm 31\;\mathrm{mT}$. The correspondence between coercive field and field of maximum resistance proves that the alignment of the clusters’ magnetic moments causes the resistivity of the sample to decrease.

We emphasize that the magnetic properties measured with SQUID magnetometry correspond to the full ensemble of particles on the sample, while the transport measurements only correspond to the film between the two voltage sensing contact lines. However, a clear correlation between the measured magnetization and the observed saturating magnetoresistance can be noted as expected. In particular, below the blocking temperature the maxima in resistance occur at the coercive fields, as is shown in Figure 11.

Also saturation fields correspond well to each other for both types of measurement. 

The reduction in saturation magnetization as temperature increases, is thus at least in part responsible for the observed reduction in magnetoresistance with increasing temperature. The comparatively stronger reduction in magnetoresistance with temperature, can be explained by increased conduction through the matrix. 

#### 3.3.3. Percolation in Fe-Ge Nanocomposite Films

**Figure 12:**
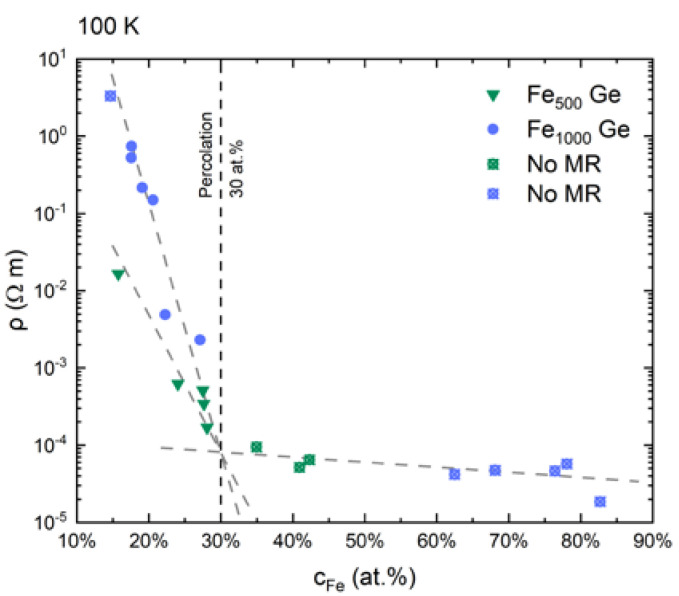
Resistivity at 100 K vs. Fe concentration of Fe-Ge nanocomposite films. Green triangles represent nanocomposites containing Fe_500_, blue circles those containing Fe_1000_. At concentrations above 30 at.% the resistivity’s order of magnitude is in accordance with minimum metallic conduction [51]. Below, resistivity strongly increases with decreasing concentration since conducting paths break up into isolated chains of agglomerating clusters between which the charge carriers must travel through the matrix. The change between these two conduction regimes appears as a kink in the resistivity vs. concentration plot. At 100 K the resistivity of a-Ge is of the order of $10^{4}\;\Omega m$ [52].

Embedding Fe clusters into an a-Ge matrix at varying concentration made it possible to tune the nanocomposites’ resistivity over five orders of magnitude. Plotting resistivity vs. concentration data, as shown in Figure 12, exhibits a kink at $c_{Fe}=30\;\mathrm{at}.\%$ which we relate to a change between two regimes of conduction [50]: On the right hand side of the kink the sample resistivity changes only little with further increased Fe concentration from $10^{-4}\;Ωm$ to $10^{-5}\;Ωm$ in accordance with the minimum metallic conductivity [51].

In this metallic (percolation) regime transport happens through metallic channels [53]. The crossed symbols indicate that nanocomposites with $c_{Fe}>30\;\mathrm{at}.\%$ did not show any magnetoresistive behavior within the sensitivity of our equipment. Below the kink resistivity starts to increase strongly with decreasing Fe concentration and samples start to show magnetoresistive behavior. In this transition towards the dielectric regime series of agglomerated but isolated chains of clusters rather than connected pathways define charge carrier transport [53]. This confirms the 3D expansion of the 2D structure observed in the EFTEM analysis shown in Figure 8. 

The critical volume percentages required to form closed paths through randomly occupied sites in sc, bcc, fcc, hcp and diamond lattices of hard spheres were found to be quite similar and 15.4 vol.% on average [54]. This value holds for any 3D system, i.e., also amorphous ones, because any arrangement of atoms can be approximated to one of the mentioned lattices [55]. With bulk densities (see Appendix A) this threshold corresponds to 27 at.% for Fe-Ge nanocomposites. This is in good agreement with the observed value of 30 at.%.

## 4. Discussion

### 4.1. Tunneling in Fe-Ge Nanocomposites

The discussion is started by investigating the conduction mechanism by comparing the temperature dependence of resistivity to theoretical models. We find evidence that tunneling tends to dominate below a temperature of about 100 K as the conductivity of the semiconducting matrix via variable range hopping becomes less important. 

Between isolated conducting clusters, transport is generally by tunneling [56]. In case of a-Ge barriers conduction via variable range hopping (VRH) is also possible [57,58]. VRH is the dominant process of transport in a-Ge at room temperature and below [52,59,60] and exhibits a characteristic $\log\left(\rho /T^{1/2}\right)\propto T^{-1/4}$ resistivity vs. temperature dependence. The base units of a-Ge are distorted tetrahedrons [61]. These form a covalent network in which not all the valence electrons of the Ge atoms have another one near enough, to form a covalent bond. These remain unbound corresponding to a dangling bond. These dangling bond states have their energy close to the Fermi level [45,62] and it is these valences which promote VRH conduction. We confirmed the temperature dependence of resistance in a pure a-Ge sample. The characteristic $T^{-1/4}$ dependence was observed only in restricted intervals at temperatures approximately above 100 K. This indicates that closer to room temperature other thermally activated mechanisms contribute to conduction. 

Treating the a-Ge as an insulator, no conduction via the matrix is possible. Then, conduction between non-touching conductive inclusions can only happen via tunneling [55]. With its pure resistivity much higher than when interspersed with Fe clusters, a-Ge can be seen as an insulator to first approximation. 

A major difference between classic tunneling, from one metallic bulk electrode across a thin tunneling barrier to another electrode, and tunneling transport in an ensemble of isolated metallic grains is that charge neutrality is broken in the latter case. This is because the tunneling of an electron turns a pair of neutral grains into a pair of oppositely singly charged grains [63]. Therefore, clusters can become Coulomb blocked. In the case of a classic tunneling barrier of width s and height ϕ this situation can be modelled by an energy $E_{C}=e^{2}/4\pi \varepsilon_{0}\varepsilon_{r}d$ required to charge a sphere of diameter d in a dielectric with relative permittivity $\varepsilon_{r}$. e and $\varepsilon_{0}$ are the elementary charge and the dielectric constant, respectively. For a-Ge we have $\varepsilon_{r}=16$ [58], so the charging energies are 40 meV and 32 meV for Fe_500_ and Fe_1000_ clusters, respectively, which is larger than the thermal energy at room temperature $k_{B}T=26\;\mathrm{meV}$. Finding the optimal conduction path through a sample becomes an optimization problem. Carriers are less likely to tunnel to smaller grains because of the higher charging energy. Similarly, they avoid larger grains with lower charging energies but located at larger tunneling distance. We assume that $sE_{C}=const.$ since variations in cluster size are mostly due to agglomeration [51,64]: Agglomerates leave behind larger gaps in their surroundings. The carriers will therefore follow paths via grains with similar charging energies, which is the path of highest mobility. This results in a resistivity given by

(1) ρ(T)=ρ0 exp(2(CkBT)1/2),

where $C=\chi sE_{C}$ with $\chi =\sqrt{2m^{*}\phi /\hbar^{2}}$ and effective carrier mass $m^{*}$ [51,64]. Corresponding resistivity data appear as a straight line in a $\log\left(\rho \right)\;\mathrm{vs}.T^{-1/2}$ scaled plot (‘tunneling scaling’) when this process is dominating. 

**Figure 13:**
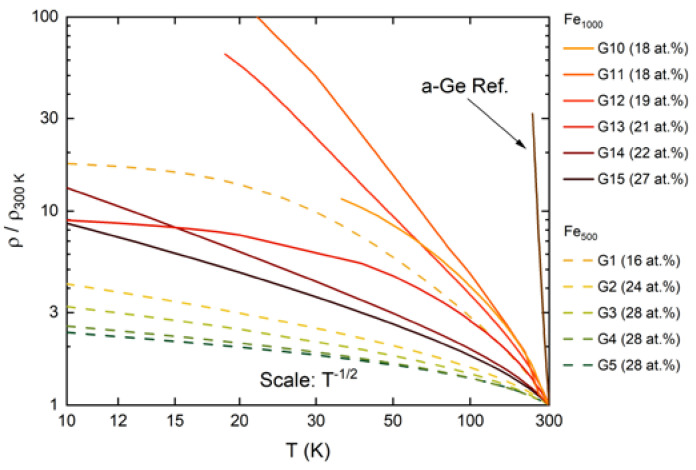
Log-scaled relative resistivity RR vs. temperature scaled as $T^{-1/2}$. Straight line appearance in this scaling is characteristic for tunneling between isolated metallic grains (see text). RR increases slower with decreasing temperature for samples with a smaller amount of Fe clusters embedded. RR is generally smaller for samples containing the smaller clusters.

Figure 13 is a revision of the Fe-Ge nanocomposite resistivity vs. temperature plot in Figure 7, but now showing the relative resistivity (RR) ρ(T)/ρ(300 K) with the axes scaled according to the ‘tunneling scaling’ introduced above.

It can be seen that for most samples the curves adopt a straight line behavior at lower temperatures. The absolute resistance of G10 was too high to be measured at sufficiently low temperature. We note that for samples G1, G10 and G13 the RR saturates as temperature decreases. As discussed before, this is most likely because of a shunt resistance that is large, but starts to dominate as the resistance of the nanocomposite increases. The shunt resistance is more or less temperature independent and is not affected by the magnetic field. This is in perfect agreement with the reducing tunneling magnetoresistance for decreasing temperature seen only for these three samples.

A more thorough analysis of the slopes of the straight-line ranges shows that the tunneling parameter C varies from 0.1 meV to 20 meV. For samples G11 and G12 it most accurately followed the temperature scaling related to tunneling.

Assuming $m^{*}=m_{e}$ and with the charging energies $E_{C}$ given in the text above, average tunneling barrier heights can be calculated from parameters C when the tunneling barrier width is chosen as s=MPS. For samples G12 and G11 this results in tunneling barrier heights ϕ=1.2 meV and ϕ=2.5 meV, respectively. This is one order of magnitude lower than experiments on a-Ge tunneling barriers revealed ($\phi_{Ge}=20\;\mathrm{meV}$), [58] and two orders of magnitude lower than half the band gap of Ge ($E_{g}/2\approx 0.35\;\mathrm{eV}$, [65]). That the latter does not apply is not surprising and merely indicates that dangling bond states cannot be neglected. The still lower barriers may be in part due to the uncertainty associated with the value of the effective mass. The value best compatible with the data in Reference [58] was found to exceed the free electron mass. Effective mass derived from heat capacity measurements point at an abnormally large effective electron mass of $8.0\;m_{e}$ for Fe [66]. However, according to Equation (1) an effective electron mass $m^{*}>m_{e}$ yields even smaller barrier heights. Finally, it is likely that a small amount of alloying of cluster atoms with the matrix atoms leads to a reduction in barrier height. 

Holdenried et al. [38] deposited and analyzed samples of well-defined nanometer sized Co clusters embedded in frozen Kr and Xe noble gas matrices of a structure comparable to our nanocomposite samples. They found a perfect straight line dependence in tunneling scaled resistivity vs. temperature plots up to the matrix elements’ melting points. However, in agreement to our observation, they deduced unphysically low tunneling distances or reduced barrier heights when using the same model. The average diameter of 4.5 nm of the Co clusters used there and concentrations between 13 vol.% and 29 vol.% result in a corresponding range of values for MPS of 2.7 nm to 1.0 nm. The MPS of the present Fe-Ge samples is in the same range. 

#### 4.1.1. Approximation as Single Barrier Junction

Just below the percolation limit it is valid to assume that conduction occurs only through a small number of tunnel barriers. Therefore, as an alternative approach addressing this limit, we model the temperature dependence of resistivity with a single barrier model. There is a clear correlation between the maximum barrier widths, derived in this way, and MPS. It is compatible with the idea that as MPS increases connected islands of clusters decrease in size. The connected islands form shunting resistances that reduce the measured tunneling magnetoresistance (see Section 4.2).

The largest gaps within a network of connected or close-by clusters, agglomerates and chains may form the bottlenecks for carrier transport and which therefore may largely influence the resistivity of a nanocomposite film. Interpreting each sample as dominated by these bottlenecks with a width $s_{max}$ and assuming only one such gap along each path of highest mobility through a sample film we can try to apply Simmons’s theory [67,68] for tunneling between ferromagnetic electrodes to the present nanocomposite samples. To calculate either a barrier’s width s or its height ϕ from another, a rearranged version of the low-voltage limit with a Stratton-like [69] temperature dependence γ(T) [70] is used. This is justified by the linear I-V characteristics we found for our nanocomposites.

(2) $$R\left(T\right)={\left(\alpha \;\gamma \left(T\right)\;S\right)}^{-1},$$

with $\alpha =e^{2}A\sqrt{\phi }/4\pi hs^{2}\;\exp\left(-A\sqrt{\phi }\right)$, $\gamma \left(T\right)=\pi Bk_{B}T/\sin\left(\pi Bk_{B}T\right)$, $A=\left(4\pi s/h\right)\sqrt{2m^{*}}$ and $B=A/2\sqrt{\phi }$, $m^{*}$ the effective electron mass and R(T) a sample film’s resistance at a temperature T within the range where tunneling is dominating. S is the area fraction of a nanocomposite layer’s cross-sectional area that is taken up by the cross-sections of the clusters $\pi d_{Cl}^{2}/4$.

**Figure 14:**
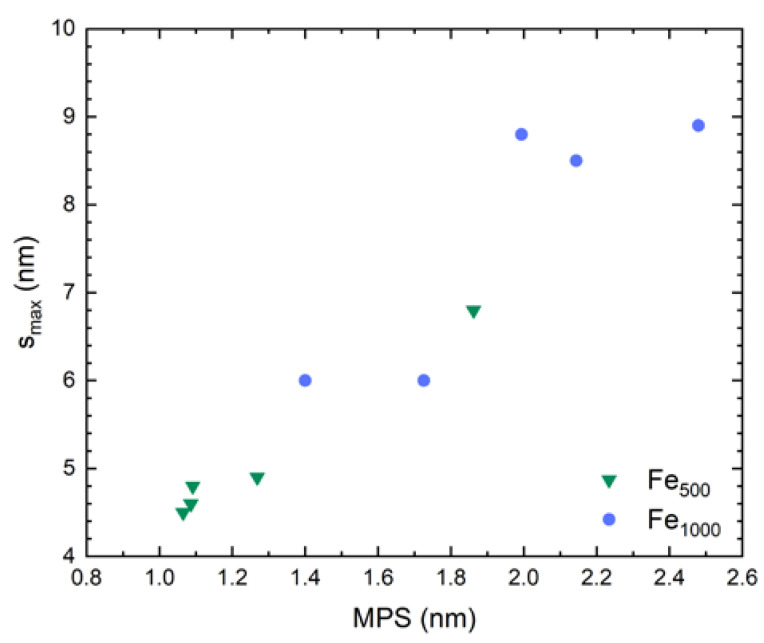
Estimated bottleneck tunneling barrier widths in case conduction along each path is dominated by one single gap.

For the two samples showing the plateau in tunneling scaling, G12 and G11, we observe bottleneck barrier widths $s_{max}$ of 8 nm and 9 nm, respectively, when $\phi_{Ge}=20\;\mathrm{meV}$ [58] is used. A summary of all samples’ bottleneck widths is shown in Figure 14.

There is an approximately linear relation of $s_{max}$ with MPS. Values of up to 10 nm are reasonable, since this is larger than the average cluster separations, as expected. Furthermore, it corresponds to the barrier width above which VRH was found to become the leading transport process in other studies on tunneling through a-Ge barriers [58]. Since Gibson et al. [58] found their data are fitted best when an effective electron mass $m^{*}=2.8\;m_{e}$ is used we also used this value to calculate the results shown. 

### 4.2. Correlation between Resistivity, Tunneling Magnetoresistance and Mean Particle Separation

In this section the dependence of resistance and tunneling magnetoresistance on the average distance between the surfaces of two neighboring clusters MPS is investigated. As defined above, the latter is calculated by finding the distance between cluster surfaces if the clusters were arranged in a simple cubic lattice at the same concentration (see Appendix A). In Section 4.1.1 we showed that even if we assume that the conduction in the nanocomposite films is dominated by one largest gap, the derived gaps are not unreasonably large and correlate as expected with MPS. We expect there to be a continuous transition between the latter picture of dominating bottlenecks and that of conduction via well separated single clusters, but MPS remains a good scaling parameter in both regimes.

**Figure 15:**
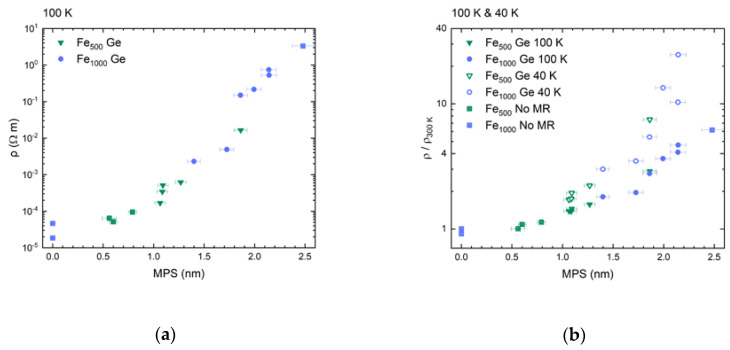
(**a**) Resistivity at 100 K and (**b**) relative resistivity at 100 K and 40 K vs. mean particle separation MPS. Both physical quantities exhibit an exponential dependence on MPS. Crossed symbols represent samples not showing magnetoresistive behavior at 100 K.

Accordingly, we find that both resistivity and tunneling magnetoresistance scale with MPS, too, as is to be expected for temperatures at which tunneling dominates. In Figure 15a we show resistivity data at 100 K as a function of MPS.

We find that nanocomposites resistivity increases with MPS. This is not the case, for example, if plotted against the mean distance between cluster centers. Within the limits of uncertainty we therefore find no dependence on clusters size. Resistivity increases exponentially with MPS. We interpret this as a clear sign of increasing average tunneling barrier widths as well as an increase in number of barriers. 

If conduction in the matrix dominated, the increase would be less significant. It also agrees with the literature, where VRH was found to dominate in a-Ge tunneling junctions with barriers wider than 10 nm [58]—much larger than the MPS in the present experiment. However, because of the random distribution of clusters and the associated variation in present barrier widths, we assume both VRH and tunneling to occur in the present samples. Moreover, VRH as the intrinsic process of carrier transport of a-Ge, therefore, is the low concentration, that is, large barrier width limit of the nanocomposite. With increasing Fe content distances between conducting inclusions decrease and tunneling becomes more probable. 

We find no clear dependence on cluster size for RR, too, which is shown at 100 K (solid symbols) and 40 K (open symbols) in Figure 15b. Representing a cross-section through Figure 13 at these very temperatures, Figure 15b again evidences the resistivity’s increasingly stronger low temperature rise with increasing MPS. We attribute this to a progressively rising fraction of Coulomb blocked clusters [71]. 

**Figure 16:**
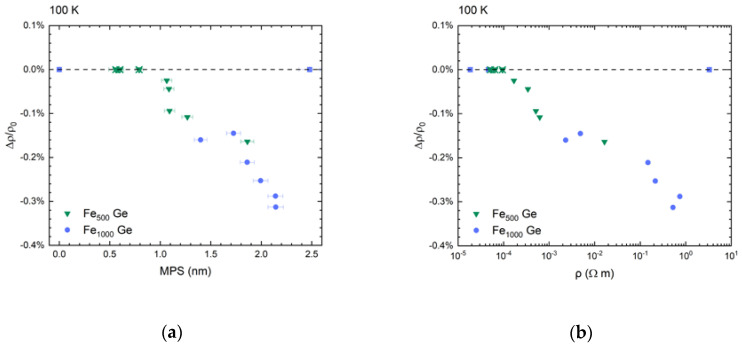
(**a**) Tunneling magnetoresistance vs. mean particle separation and (**b**) vs. resistivity. The shown data were recorded at 100 K. Crosses symbolize samples not showing magnetoresistive behavior.

Plotting the nanocomposites’ tunneling magnetoresistance components versus MPS in Figure 16a depicts the effect’s dependence on the spacing between cluster surfaces.

It suggests that only cluster surface states participate in tunneling processes. The correlation of a nanocomposite’s resistivity and its tunneling magnetoresistance is shown in Figure 16b. 

On the lower MPS side in Figure 16a it is clear that a minimum separation of about 0.8 nm is required for tunneling magnetoresistance to appear, which corresponds to the percolation threshold discussed above. The visible increase in tunneling magnetoresistance with increasing MPS and its rather small overall value can be understood using the following simple parallel resistor model. 

As mentioned above, the remaining connected islands below the percolation threshold form shunting resistances that are not field dependent. Their effect can be accumulated into a single parallel resistor with an increasing resistivity $\rho_{S}$ as MPS increases. With the presence of this shunting resistance the measured tunneling magnetoresistance ${\left(\Delta \rho /\rho_{0}\right)}_{shunted}$ can be expressed by

(3) $${\left(\frac{\Delta \rho }{\rho_{0}}\right)}_{shunted}=\frac{1}{1+\;\frac{\rho_{0}^{\prime }}{\rho_{S}}\left(1+{\left(\frac{\Delta \rho^{\prime }}{\rho_{0}^{\prime }}\right)}_{no\;shunt}\right)}\;{\left(\frac{\Delta \rho^{\prime }}{\rho_{0}^{\prime }}\right)}_{no\;shunt}.$$

Here, $\rho_{0}$ and Δρ are the resistivity of the shunted nanocomposite at zero field and its change due to an applied magnetic field. $\rho_{0}^{\prime }$ and $\Delta \rho^{\prime }$ are the corresponding quantities for the pure nanocomposite with magnetoresistance ${\left(\Delta \rho^{\prime }/\rho_{0}^{\prime }\right)}_{no\;shunt}$. For the case $\rho_{S}\gg \rho_{0}^{\prime }$ the measured magnetoresistance corresponds well to the magnetoresistance measured without the effect of the shunt. For $\rho_{S}\ll \rho_{0}^{\prime }$ the observed magnetoresistance approaches zero, as is the case above the percolation threshold. At zero temperature tunneling magnetoresistance is determined by the spin polarization P and can be calculated using the relation $TMR_{0}=-P^{2}/\left(1+P^{2}\right)$ [22,38,72]. For Fe, a spin polarization of 43% is observed in superconducting tunneling experiments. Similar values of spin polarization are observed from various superconductor/Fe point contact spectroscopy experiments [73]. Hence, values as large as $TMR_{0}=-15.6\%$ are possible. Considering this and the here observed maximal values of tunneling magnetoresistance of about −0.5% at the lowest temperatures, we can deduce the relative shunting resistivity to be $\rho_{S}/\rho_{0}^{\prime }\approx 0.028$. This means only about 3% of the excitation current contributes to the tunneling magnetoresistance. 

Thus, the low value observed here can be associated with conduction through connected networks of clusters that is field independent. At high temperatures we expect that the matrix increasingly contributes to lowering the shunting resistance. At the low temperature end we expect that the resistance on the nanocomposite has increased so much, that other shunting resistances due to contamination between contact lines start to dominate. Additionally, as MPS increases VRH through the a-Ge matrix becomes non-negligible as well [58]. The result is that simply increasing MPS cannot yield larger values for tunneling magnetoresistance in the present system, too. Evidence for this is the negligible tunneling magnetoresistance found in sample G9. Furthermore, increased MPS implies larger resistivity, the measurement of which was limited by the available instruments.

A possible way out of this dilemma could be a different approach of depositing the nanocomposite: Instead of continuous co-deposition, clusters and matrix can also be deposited in alternating order. This way a monolayer of clusters could be fully coated with matrix material before the next layer is deposited. Clearly, choosing a matrix material that wets the cluster surface would be beneficial in either of the two approaches. 

Venugopal et al. [74,75] produced Fe-Ge cluster–matrix films by implanting Fe ions into a crystalline Ge wafer. There, the process of implantation damaged their matrix, turning it partially amorphous. This makes it relevant to the present study to a certain extent. In their samples, Fe clusters with an average diameter of 4 nm formed from the precipitation of Fe. Applying a parallel resistor model they extracted a magnetoresistance of −19% at 180 K for their film containing an average concentration of 23 at.%. Within a field range of ±800 mT the shape of their magnetoresistance curves is similar to the one shown here in Figure 11a. However, Venugopal et al. [74,75] prepared films with mainly crystalline p-type doped matrix and, therefore, attribute the observed negative magnetoresistance to spin-dependent scattering of charge carriers by magnetic iron clusters, i.e., the granular GMR effect. We do not expect the saturating granular GMR effect in the presented nanocomposites. This is because we observed the resistivity to strongly depend on the mean particle separation of a film and because there is no transport via carriers thermally excited into a conduction band. 

### 4.3. Influence of the A-Ge Matrix on Tunneling and Field-Dependent Magnetoresistance Components

#### 4.3.1. The Dampening Effect of Transport via Matrix States

There is a sudden change in the slope of the high field magnetoresistance for the Fe_1000_-Ge nanocomposite, as can be seen in Figure 6b. Together with the elsewise steady increase toward larger positive slope, this suggests that more than one effect determines the high field magnetoresistance of the Fe-Ge nanocomposites. 

Pure a-Ge is characterized by an anomalous negative magnetoresistance, which first increases in magnitude with decreasing temperature, then starts to decrease again, reaches zero at about 80 K and remains positive at lower temperatures [76,77]. In contrast to tunneling magnetoresistance between ferromagnetic particles, a-Ge does not get its magnetoresistance from the degree of alignment of the charge carriers’ spins, but from the change of spin relaxation times. These determine the ratio of hops requiring a spin-flip to hops which do not. The jump in the magnetoresistance slope of our samples appears at roughly the same temperature where a-Ge changes its sign of magnetoresistance. This suggests that the magnetoresistance of the a-Ge matrix at least partially influences the nanocomposite’s one. 

#### 4.3.2. Field-Dependent Magnetoresistance Effects

**Figure 17:**
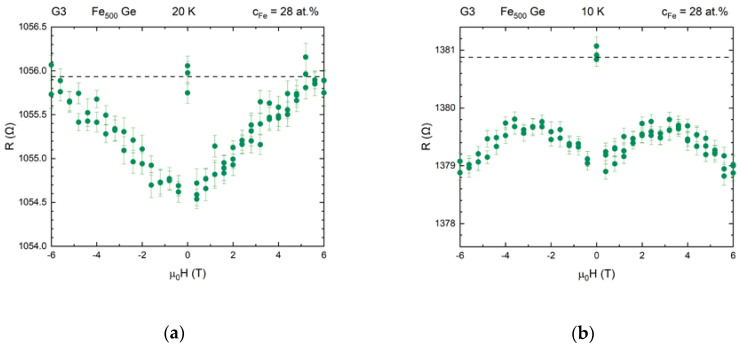
Absolute resistance vs. applied magnetic field at constant temperature. (**a**) At 20 K sample G3 shows an almost-linear in field positive magnetoresistance. (**b**) At 10 K a stronger negative effect is superimposed and changes the slope to negative at about 3 T. Such behavior was observed for most samples.

The positive magnetoresistance observed at temperatures below 90 K, as depicted in Figure 17a for sample G3 at 20 K, can have several reasons.

Non-saturating linear positive magnetoresistance (LPMR) due to microscopic conductance fluctuations can appear [14] which in our nanocomposites may be related to cluster agglomerates and the clusters’ random deposition. 

Another possible effect is due to metallic conduction within clusters and agglomerations of these. In a ferromagnet the 4s sub-bands are spin-split because of the net moment of 3d electrons [78]. The gap Δ(0) by which these sub-bands are generically split is further increased by a Zeeman term that adds when an external magnetic field $\Delta \left(H\right)=\Delta \left(0\right)+g\mu_{B}H$, where Δ(0)>0.1 eV [79], $g\mu_{B}H\;\ll \;\Delta \left(0\right)$ and $k_{B}T\ll \Delta \left(H\right)$, is applied. This adds a contribution δρ to the resistivity of a film, which is linear in H both in 2D and 3D films. Gerber et al. [78], who examined Fe and Ni thin films between 3 nm and 300 nm in fields up to 60 T, explicitly mention they also observed LPMR in granular ferromagnet-semiconductor composites. To their knowledge, this is the only effect that provides a linear positive field dependence. 

The effective distribution in size of magnetic moments and large variations in the clusters’ separations affect the clusters to change between ferromagnetic and superparamagnetic state at different temperatures within the same sample. Interaction between these results in a negative field-dependent magnetoresistance [80,81,82,83,84]. This may explain the negative field-dependent component adding at 10 K for most samples, one of which is shown in Figure 17b. 

Our nanocomposite films did not show any magnetoresistance above percolation limit, when in the granular metal regime. Therefore, the field-dependent magnetoresistances is not caused by a wide-spread 3D arrangement of touching metallic grains. 

## 5. Conclusions

We have combined semiconducting and ferromagnetic properties in a nanocomposite by applying size-selective cluster ion beam deposition. The precise control over the nanocomposites’ composition allowed us to control concentration and cluster size independently. In this case study, we co-deposited Fe clusters containing either 500±10% or 1000±10% atoms per cluster and amorphous Ge from an effusion cell. We measured temperature-dependent resistivity and magnetoresistance of the resulting nanocomposite films in a temperature range from room temperature down to 40 K (and lower in some cases) and in magnetic fields up to 6 T.

The samples showed a saturating negative magnetoresistance, which we identified as tunneling magnetoresistance. It is superimposed by at least one field-dependent contribution. Resistivity and tunneling magnetoresistance were found to correlate with cluster concentration, and thus the average distance between the surfaces of neighboring clusters. No dependence on the cluster size was found after separating out the associated change in the latter surface-to-surface distances.

To better characterize the tunneling conduction we applied a model for tunneling via isolated metallic grains and also approximated paths of conduction through the nanocomposite as being dominated by the largest bottleneck gaps. In both cases we find reasonable agreement with related reports in literature. 

The observed magnetoresistance effects in the prepared nanocomposite films are less than 0.5% in magnitude. We argue that this is mainly due to a low shunting resistance of the remaining islands of connected clusters, which effectively reduces the measured tunneling magnetoresistance. At cluster concentrations close to the percolation threshold, the formation of these islands cannot be fully avoided. This is the case even when surface diffusion of both clusters and matrix atoms is completely suppressed. A reduction of the cluster concentration is not an option, due to the expected increased contribution of conduction through the matrix, which is equally expected to reduce the measured tunneling magnetoresistance. 

However, both a further reduction of the substrate temperature and an increase of the cluster deposition energy (to achieve pinning of the clusters), could help to further reduce the formation of the shunting cluster assemblies. A different promising approach would be to alternately deposit clusters and matrix instead of continuous co-deposition. Once the problem of low tunneling magnetoresistance is solved, perhaps also by choosing a different semiconductor as matrix, it will be possible to confirm the absence of a cluster size effect. 

## Figures and Tables

**Table 1:** Exemplary process parameters for the two cluster species with diameter $d_{\mathrm{Cl}}$. Ar & He gas flow $q_{\mathrm{Ar}}/q_{\mathrm{He}}$, AC pressure $p_{\mathrm{AC}}$, sputter power $P_{\mathrm{Sputter}}$, AC temperature $T_{\mathrm{AC}}$, beam potential $U_{\mathrm{beam}}$, sector magnet field B and resulting negative cluster ion current $I_{\mathrm{Cl}}$ of the size-filtered total beam measured with a Faraday cup right behind the sector magnet. The cluster ions were soft-landed with a kinetic energy of 56 eV. For the cluster diameters see Appendix A.

Parameter Cluster	$d_{Cl}$ (nm)	$q_{Ar}/q_{He}$ (sccm)	$p_{AC}$ (mbar)	$P_{Sputter}$ (W)	$T_{AC}$ (K)	$U_{beam}$ (V)	B (T)	$I_{Cl}$ (pA)
Fe_500_	2.2	100/300	2.3	45	100	+400	0.97	115
Fe_1000_	2.8	125/110	1.9	70	100	+400	1.36	100

**Table 2:** Fe_500_-Ge and Fe_1000_-Ge nanocomposite samples listed by their sample ID, Fe concentration, layer thickness and average cluster surface to surface distance. Samples with their ID in parentheses did not show magnetoresistive behavior since they were too high or too low in concentration. The samples are listed by Fe concentration in ascending order.

Sample ID Fe_500_ Ge	$c_{Fe}$ (at.%)	t (nm)	ρ at 100 K (Ωm)	MPS (nm)	TMR at 100 K (%)
G1	16	232	1.6 × 10^−2^	1.9	−0.16
G2	24	120	6.2× 10^−4^	1.3	−0.11
G3	28	130	5.1× 10^−4^	1.1	−0.09
G4	28	121	3.4× 10^−4^	1.1	−0.04
G5	28	17	1.7× 10^−4^	1.1	−0.03
(G6)	35	102	9.5× 10^−5^	0.8	0
(G7)	41	52	5.2× 10^−5^	0.6	0
(G8)	42	68	6.5× 10^−5^	0.6	0
**Fe_1000_ Ge**					
(G9)	15	197	3.3 × 10^0^	2.5	0
G10	18	523	7.4 × 10^−1^	2.1	−0.29
G11	18	340	5.3 × 10^-1^	2.1	−0.31
G12	19	148	2.2 × 10^−1^	2.0	−0.25
G13	21	255	1.5 × 10^−1^	1.9	−0.21
G14	22	167	4.9 × 10^−3^	1.7	−0.15
G15	27	234	2.3 × 10^−3^	1.4	−0.16
(G16)	76	70	4.6 × 10^−5^	0 ^1^	0
(G17)	83	36	1.9 × 10^−5^	0 ^1^	0

^1^ Sample is above percolation limit.

## Appendix A. Calculation of the Mean Particle Separation

The 20 keV beam used for EDX mapping is assumed to deeply enter the substrate so that a linear response independent from deposition depth is assumed for the deposited film layers. With the crystal balance recorded thicknesses of Ge $t_{B}$, $t_{M}$ and $t_{P}$ for buffer, matrix and protection layer, respectively, and the EDX determined atom concentration ratio x the atom concentration in the nanocomposite film is

(A1) $$c_{Fe}=1-\frac{\left(1-x\right)\;t_{M}}{x\;\left(t_{B}+t_{P}\right)+t_{M}}$$

and the film thickness is

(A2) $$t=t_{M}\;\left(1+\frac{c_{Fe}}{1-c_{Fe}}\times \frac{m_{at}^{Fe}}{m_{at}^{Ge}}\times \frac{\rho_{Ge}}{\rho_{Fe}}\right).$$

From the volume concentration

(A3) $$c_{vol}={\left(1+\frac{1-c_{Fe}}{c_{Fe}}\times \frac{m_{at}^{Ge}}{m_{at}^{Fe}}\times \frac{\rho_{Fe}}{\rho_{Ge}}\right)}^{-1}$$

the mean particle separation MPS (average distance between the surfaces of neighboring clusters) can be calculated by assuming packing density of a simple cubic arrangement of the clusters of π/6≈0.524:

(A4) MPS=((π6 cvol)13−1) dCl

where $m_{at}^{Fe,Ge}$ are the atomic masses of Fe and Ge, respectively. The mass density of Fe was assumed to be equal to bulk value $\rho_{Fe}=7.9\;g/{\mathrm{cm}}^{3}$ and that of a-Ge was determined to be $\rho_{Ge}=5.1\;g/{\mathrm{cm}}^{3}$ by means of XRR measurements. With the mass density of bulk Fe, the cluster diameters $d_{Cl}$ are estimated to be 2.2 nm and 2.8 nm for Fe_500_ and Fe_1000_ clusters, respectively. Possible denser random packing does not change MPS by too much.

## Appendix B. Magnetoresistance for Different Orientations of the Magnetic Field

The granular tunneling magnetoresistance effect is isotropic, that is, its magnitude does not depend on the relative alignment between magnetic field and current. This is evidenced in Figure A1 at three different temperatures (red 200 K, blue 100 K, and yellow 40 K).

The magnetoresistance curves recorded in longitudinal (solid line, magnetic field parallel to current), transverse (circle, magnetic field perpendicular to current but in film plane) and perpendicular orientation (crosses, magnetic field perpendicular to film plane) match within the limits of uncertainty. To change the orientation the sample had to be fully removed from and reinstalled to the PPMS sample holder, in particular, the wire bonding connections had to be renewed.

## References

[1] Chappert C., Fert A., Van Dau F.N. (2007). The emergence of spin electronics in data storage. Nat. Mater..

[2] Fert A., Van Dau F.N. (2019). Spintronics, from giant magnetoresistance to magnetic skyrmions and topological insulators. Comptes. Rendus. Phys..

[3] Baibich M.N., Broto J.M., Fert A., Van Dau F.N., Petroff F., Etienne P., Creuzet G., Friederich A., Chazelas J. (1988). Giant Magnetoresistance of (001)Fe/(001)Cr Magnetic Superlattices. Phys. Rev. Lett..

[4] Binasch G., Grünberg P., Saurenbach F., Zinn W. (1989). Enhanced magnetoresistance in layered magnetic structures with antiferromagnetic interlayer exchange. Phys. Rev. B.

[5] Berkowitz A.E., Mitchell J.R., Carey M.J., Young A.P., Zhang S., Spada F.E., Parker F.T., Hutten A., Thomas G. (1992). Giant magnetoresistance in heterogeneous Cu-Co alloys. Phys. Rev. Lett..

[6] Xiao J.Q., Jiang J.S., Chien C.L. (1992). Giant magnetoresistance in the granular Co-Ag system. Phys. Rev. B.

[7] Dietl T. (2010). A ten-year perspective on dilute magnetic semiconductors and oxides. Nat. Mater..

[8] Jamet M., Barski A., Devillers T., Poydenot V., Dujardin R., Bayle-Guillemaud P., Rothman J., Bellet-Amalric E., Marty A., Cibert J. (2006). High-Curie-temperature ferromagnetism in self-organized Ge1−xMnx nanocolumns. Nat. Mater..

[9] Yu S.S., Anh T.T.L., Ihm Y., Kim D., Kim H., Hong S.-K., Kim C.-S., Ryu H. Magnetic and Magnetotransport Properties of Annealed Amorphous Ge_1−*x*_Mn*_x_* Semiconductor Thin Films. Proceedings of the 2007 2nd IEEE International Conference on Nano/Micro Engineered and Molecular Systems.

[10] Sugahara S., Lee K.L., Yada S., Tanaka M. (2005). Precipitation of Amorphous Ferromagnetic Semiconductor Phase in Epitaxially Grown Mn-Doped Ge Thin Films. Jpn. J. Appl. Phys..

[11] Potzger K., Zhou S., Reuther H., Mücklich A., Eichhorn F., Schell N., Skorupa W., Helm M., Fassbender J., Herrmannsdörfer T. (2006). Fe implanted ferromagnetic ZnO. Appl. Phys. Lett..

[12] Zhou S., Potzger K., Talut G., Reuther H., von Borany J., Grötzschel R., Skorupa W., Helm M., Fassbender J., Volbers N. (2008). Fe-implanted ZnO: Magnetic precipitates versus dilution. J. Appl. Phys..

[13] Fedorchenko I.V., Aronov A.N., Marenkin S.F., Simonenko N.P., Boeva N.M., Kochura A.V., Lahderanta E. (2015). Phase diagram of the ZnSnAs_2_–MnAs system. J. Alloys Compd..

[14] Fedorchenko I.V., Kilanski L., Zakharchuk I., Geydt P., Lahderanta E., Vasilyev P.N., Simonenko N.P., Aronov A.N., Dobrowolski W., Marenkin S.F. (2015). Composites based on self-assembled MnAs ferromagnet nanoclusters embedded in ZnSnAs_2_ semiconductor. J. Alloys Compd..

[15] Myagkov V.G., Tambasov I.A., Bayukov O.A., Zhigalov V.S., Bykova L.E., Mikhlin Y.L., Volochaev M.N., Bondarenko G.N. (2014). Solid state synthesis and characterization of ferromagnetic nanocomposite Fe–In_2_O_3_ thin films. J. Alloys Compd..

[16] Liu W., Zhang H., Shi J., Wang Z., Song C., Wang X., Lu S., Zhou X., Gu L., Louzguine-Luzgin D.V. (2016). A room-temperature magnetic semiconductor from a ferromagnetic metallic glass. Nat. Commun..

[17] Chen N., Fang K., Zhang H., Zhang Y., Liu W., Yao K., Zhang Z. (2019). Amorphous magnetic semiconductors with Curie temperatures above room temperature. J. Semicond..

[18] Chen L., Yang X., Yang F., Zhao J., Misuraca J., Xiong P., von Molnár S. (2011). Enhancing the Curie Temperature of Ferromagnetic Semiconductor (Ga,Mn)As to 200 K via Nanostructure Engineering. Nano Lett..

[19] Hai P.N., Ohya S., Tanaka M. (2010). Long spin-relaxation time in a single metal nanoparticle. Nat. Nanotech..

[20] Temple R.C., McLaren M., Brydson R.M.D., Hickey B.J., Marrows C.H. (2016). Long spin lifetime and large barrier polarisation in single electron transport through a CoFe nanoparticle. Sci. Rep..

[21] Yakushiji K., Ernult F., Imamura H., Yamane K., Mitani S., Takanashi K., Takahashi S., Maekawa S., Fujimori H. (2004). Enhanced spin accumulation and novel magnetotransport in nanoparticles. Nat. Mater..

[22] Julliere M. (1975). Tunneling between ferromagnetic films. Phys. Lett. A.

[23] Fujimori H., Mitani S., Ohnuma S. (1995). Tunnel-type GMR in metal-nonmetal granular alloy thin films. Mat. Sci. Eng. B.

[24] Hirohata A., Takanashi K. (2014). Future perspectives for spintronic devices. J. Phys. D Appl. Phys..

[25] Gingrich E.C., Niedzielski B.M., Glick J.A., Wang Y., Miller D.L., Loloee R., Pratt W.P., Birge N.O. (2016). Controllable 0–π Josephson junctions containing a ferromagnetic spin valve. Nat. Phys..

[26] Weides M., Kemmler M., Kohlstedt H., Waser R., Koelle D., Kleiner R., Goldobin E. (2006). 0−π Josephson Tunnel Junctions with Ferromagnetic Barrier. Phys. Rev. Lett..

[27] Schneider M.L., Donnelly C.A., Russek S.E., Baek B., Pufall M.R., Hopkins P.F., Dresselhaus P.D., Benz S.P., Rippard W.H. (2018). Ultralow power artificial synapses using nanotextured magnetic Josephson junctions. Sci. Adv..

[28] Ioffe L.B., Geshkenbein V.B., Feigel’man M.V., Fauchère A.L., Blatter G. (1999). Environmentally decoupled sds -wave Josephson junctions for quantum computing. Nature.

[29] Yamashita T., Tanikawa K., Takahashi S., Maekawa S. (2005). Superconducting π Qubit with a Ferromagnetic Josephson Junction. Phys. Rev. Lett..

[30] Lutsev L.V., Stognij A.I., Novitskii N.N. (2009). Giant magnetoresistance in semiconductor/granular film heterostructures with cobalt nanoparticles. Phys. Rev. B.

[31] Lutsev L.V., Shelukhin L.A., Stognij A.I., Novitskii N.N. (2019). Relaxation processes of the light-induced giant injection magnetoresistance in semiconductor/granular-film heterostructures with cobalt nanoparticles. Phys. Rev. B.

[32] Takiguchi K., Anh L.D., Chiba T., Koyama T., Chiba D., Tanaka M. (2019). Giant gate-controlled proximity magnetoresistance in semiconductor-based ferromagnetic–non-magnetic bilayers. Nat. Phys..

[33] Benel C., Reisinger T., Kruk R., Hahn H. (2018). Cluster-Assembled Nanocomposites: Functional Properties by Design. Adv. Mater..

[34] Jamet M., Wernsdorfer W., Thirion C., Mailly D., Dupuis V., Mélinon P., Pérez A. (2001). Magnetic Anisotropy of a Single Cobalt Nanocluster. Phys. Rev. Lett..

[35] Oyarzún S., Domingues Tavares de Sa A., Tuaillon-Combes J., Tamion A., Hillion A., Boisron O., Mosset A., Pellarin M., Dupuis V., Hillenkamp M. (2013). Giant magnetoresistance in cluster-assembled nanostructures: On the influence of inter-particle interactions. J. Nanopart. Res..

[36] Marenkin S.F., Izotov A.D., Fedorchenko I.V., Novotortsev V.M. (2015). Manufacture of magnetic granular structures in semiconductor-ferromagnet systems. Russ. J. Inorg. Chem..

[37] Batlle X., Labarta A. (2002). Finite-size effects in fine particles: Magnetic and transport properties. J. Phys. D Appl. Phys..

[38] Holdenried M., Hackenbroich B., Micklitz H. (2001). Systematic studies of tunneling magnetoresistance in granular films made from well-defined Co clusters. J. Magn. Magn. Mater..

[39] Fischer A.S. (2015). Crystalline and Amorphous Cluster-Assembled Nano-Materials, Synthesized with a Novel Cluster Deposition System. Ph.D. Thesis.

[40] Fischer A., Kruk R., Wang D., Hahn H. (2015). Magnetic properties of iron cluster/chromium matrix nanocomposites. Beilstein. J. Nanotechnol..

[41] Fischer A., Kruk R., Hahn H. (2015). A versatile apparatus for the fine-tuned synthesis of cluster-based materials. Rev. Sci. Instrum..

[42] Haberland H., Mall M., Moseler M., Qiang Y., Reiners T., Thurner Y. (1994). Filling of micron-sized contact holes with copper by energetic cluster impact. J. Vac. Sci. Technol. A.

[43] Khare C., Gerlach J.W., Weise M., Bauer J., Höche T., Rauschenbach B. (2011). Growth temperature altered morphology of Ge nanocolumns. Phys. Status Solidi.

[44] Walley P.A., Jonscher A.K. (1968). Electrical conduction in amorphous germanium. Thin Solid Films.

[45] Mehra R.M., Shyam R., Mathur P.C. (1983). Magnetoresistance in amorphous semiconductors. Thin Solid Films.

[46] Padhi P.C., Tripathi G.S., Misra P.K. (2009). Theory of Magneto Resistance of Amorphous Semiconductors. Int. J. Mod. Phys. B.

[47] Mell H., Stuke J. (1970). Negative magnetoresistance of amorphous semiconductors. J. Non-Cryst. Solids.

[48] Coey J.M.D. (2010). Magnetism and Magnetic Materials.

[49] Westerstrand B., Nordblad P., Nordborg L. (1975). The Magnetocrystalline Anisotropy Constants of Iron and Iron-silicon Alloys. Phys. Scr..

[50] Grimaldi C. (2014). Theory of percolation and tunneling regimes in nanogranular metal films. Phys. Rev. B.

[51] Abeles B., Sheng P., Coutts M.D., Arie Y. (1975). Structural and electrical properties of granular metal films. Adv. Phys..

[52] Kubelík I., Tříska A. (1972). The temperature dependence of the electrical conductivity in amorphous germanium. Czech J. Phys..

[53] Sheng P. (1992). Feature article: Electronic transport in granular metal films †. Philos. Mag. B.

[54] Scher H., Zallen R. (1970). Critical Density in Percolation Processes. J. Chem. Phys..

[55] Balberg I. (2009). Tunnelling and percolation in lattices and the continuum. J. Phys. D Appl. Phys..

[56] Sheng P. (1980). Fluctuation-induced tunneling conduction in disordered materials. Phys. Rev. B.

[57] Mott N.F. (1969). Conduction in non-crystalline materials. Localized states in a pseudogap and near extremities of conduction and valence bands. Philos. Mag..

[58] Gibson G.A., Meservey R. (1985). Properties of amorphous germanium tunnel barriers. J. Appl. Phys..

[59] Hauser J.J., Staudinger A. (1973). Electrical and Structural Properties of Amorphous Germanium. Phys. Rev. B.

[60] Morgan M., Walley P.A. (1971). Localized conduction processes in amorphous germanium. Philos. Mag..

[61] Richter H., Breitling G. (1958). Struktur des amorphen Germaniums und Siliciums. Z. für Nat. A.

[62] Rockett A. (2008). The Materials Science of Semiconductors.

[63] Mitani S., Fujimori H., Ohnuma S. (1997). Spin-dependent tunneling phenomena in insulating granular systems. J. Magn. Magn. Mater..

[64] Sheng P., Abeles B., Arie Y. (1973). Hopping Conductivity in Granular Metals. Phys. Rev. Lett..

[65] Sze S.M., Ng K.K. (2007). Physics of Semiconductor Devices.

[66] Ashcroft N.W., Mermin N.D. (2013). Festkörperphysik—4. verb. Aufl..

[67] Simmons J.G. (1963). Generalized Formula for the Electric Tunnel Effect between Similar Electrodes Separated by a Thin Insulating Film. J. Appl. Phys..

[68] Simmons J.G. (1963). Low-Voltage Current-Voltage Relationship of Tunnel Junctions. J. Appl. Phys..

[69] Stratton R. (1962). Volt-current characteristics for tunneling through insulating films. J. Phys. Chem. Solids.

[70] Simmons J.G. (1964). Generalized Thermal *J-V* Characteristic for the Electric Tunnel Effect. J. Appl. Phys..

[71] Schelp L.F., Fert A., Fettar F., Holody P., Lee S.F., Maurice J.L., Petroff F., Vaurès A. (1997). Spin-dependent tunneling with Coulomb blockade. Phys. Rev. B.

[72] Meservey R., Tedrow P.M. (1994). Spin-polarized electron tunneling. Phys. Rep..

[73] Soulen R.J. (1998). Measuring the Spin Polarization of a Metal with a Superconducting Point Contact. Science.

[74] Venugopal R., Sundaravel B., Cheung W.Y., Wilson I.H., Wang F.W., Zhang X.X. (2001). Magnetic properties of nanoclusters formed by implantation of Fe into Ge using a metal-vapor vacuum arc ion source. Phys. Rev. B.

[75] Venugopal R., Sundaravel B., Wilson I.H., Wang F.W., Zhang X.X. (2002). Structural and magnetic properties of Fe–Ge layer produced by Fe ion-implantation into germanium. J. Appl. Phys..

[76] Kubelík I., Tříska A. (1973). Two types of magnetoresistance in amorphous germanium. Czech J. Phys..

[77] Kubelík I., Tříska A. (1973). Magnetoresistance in amorphous germanium. Czech J. Phys..

[78] Gerber A., Kishon I., Korenblit I.Y., Riss O., Segal A., Karpovski M., Raquet B. (2007). Linear Positive Magnetoresistance and Quantum Interference in Ferromagnetic Metals. Phys. Rev. Lett..

[79] Phillips J.C. (1964). Fermi Surface of Ferromagnetic Nickel. Phys. Rev..

[80] Yu T., Chen P. (2011). Abnormal Resistance and Magnetoresistance Temperature Dependence in Fe-Semiconductor Granular Films. IEEE Trans. Magn..

[81] Wiser N. (1996). Phenomenological theory of the giant magnetoresistance of superparamagnetic particles. J. Magn. Magn. Mater..

[82] Hickey B.J., Howson M.A., Musa S.O., Wiser N. (1995). Giant magnetoresistance for superparamagnetic particles: Melt-spun granular CuCo. Phys. Rev. B.

[83] Péter L., Rolik Z., Kiss L.F., Tóth J., Weihnacht V., Schneider C.M., Bakonyi I. (2006). Temperature dependence of giant magnetoresistance and magnetic properties in electrodeposited Co-Cu/Cu multilayers: The role of superparamagnetic regions. Phys. Rev. B.

[84] Bakonyi I., Péter L., Rolik Z., Kiss-Szabó K., Kupay Z., Tóth J., Kiss L.F., Pádár J. (2004). Decomposition of the magnetoresistance of multilayers into ferromagnetic and superparamagnetic contributions. Phys. Rev. B.

